# An Algorithm for Nonparametric Estimation of a Multivariate Mixing Distribution with Applications to Population Pharmacokinetics

**DOI:** 10.3390/pharmaceutics13010042

**Published:** 2020-12-30

**Authors:** Walter M. Yamada, Michael N. Neely, Jay Bartroff, David S. Bayard, James V. Burke, Mike van Guilder, Roger W. Jelliffe, Alona Kryshchenko, Robert Leary, Tatiana Tatarinova, Alan Schumitzky

**Affiliations:** 1 Laboratory of Applied Pharmacokinetics and Bioinformatics, Children’s Hospital of Los Angeles, Los Angeles, CA 90027, USA; wyamada@chla.usc.edu (W.M.Y.); mneely@chla.usc.edu (M.N.N.); dbay007@earthlink.net (D.S.B.); uphill@cox.net (M.v.G.); r.jelliffe@usc.edu (R.W.J.); alona.kryshchenko@csuci.edu (A.K.); 2 Pediatric Infectious Diseases, Children’s Hospital of Los Angeles, Keck School of Medicine, University of Southern California, Los Angeles, CA 90027, USA; 3 Department of Mathematics, University of Southern California, Los Angeles, CA 90089, USA; bartroff@usc.edu; 4 Jet Propulsion Laboratory, California Institute of Technology, Pasadena, CA 91109, USA; 5 Department of Mathematics, University of Washington, Seattle, WA 98195, USA; burke@math.washington.edu; 6 Department of Mathematics, California State University Channel Islands, University Dr, Camarillo, CA 93012, USA; 7 Certara, Raleigh, NC 27606, USA; Bob.Leary@certara.com; 8 Department of Biology, University of La Verne, La Verne, CA 91750, USA; ttatarinova@laverne.edu

**Keywords:** mixture distribution, mixture model, high dimensional statistics, nonparametric maximum likelihood, primal-dual interior-point method, adaptive grid, population model

## Abstract

Population pharmacokinetic (PK) modeling has become a cornerstone of drug development and optimal patient dosing. This approach offers great benefits for datasets with sparse sampling, such as in pediatric patients, and can describe between-patient variability. While most current algorithms assume normal or log-normal distributions for PK parameters, we present a mathematically consistent nonparametric maximum likelihood (NPML) method for estimating multivariate mixing distributions without any assumption about the shape of the distribution. This approach can handle distributions with any shape for all PK parameters. It is shown in convexity theory that the NPML estimator is discrete, meaning that it has finite number of points with nonzero probability. In fact, there are at most *N* points where *N* is the number of observed subjects. The original infinite NPML problem then becomes the finite dimensional problem of finding the location and probability of the support points. In the simplest case, each point essentially represents the set of PK parameters for one patient. The probability of the points is found by a primal-dual interior-point method; the location of the support points is found by an adaptive grid method. Our method is able to handle high-dimensional and complex multivariate mixture models. An important application is discussed for the problem of population pharmacokinetics and a nontrivial example is treated. Our algorithm has been successfully applied in hundreds of published pharmacometric studies. In addition to population pharmacokinetics, this research also applies to empirical Bayes estimation and many other areas of applied mathematics. Thereby, this approach presents an important addition to the pharmacometric toolbox for drug development and optimal patient dosing.

## 1. Introduction

Pharmacokinetic studies in healthy volunteers commonly collect multiple observations in each subject. These datasets often arise from Phase I clinical trials and have been traditionally analyzed by noncompartmental methods or by modeling via the standard two-stage approach. Patient datasets often contain complex dosage regimens and only a few (or one) observation(s) per patient. To analyze such sparse datasets, e.g., in pediatric or critically ill patients, population modeling is required, since noncompartmental analysis and the standard two-stage approach are not applicable [1,2]. Population modeling has been shown to estimate PK parameters without bias and with good precision for such sparse datasets [3]. Parametric population modeling algorithms are commonly used and assume typically either normal or log-normal distributions for the between subject variability of PK parameters. While this assumption is made for virtually every parametric population PK model, it is difficult to prove, especially for datasets with a small number of subjects. Nonparametric population modeling can describe a multivariate distribution of PK parameters without assuming any shape of the PK parameter distribution. This is a key advantage of the nonparametric approach that is based on the exact log-likelihood. The present work comprehensively describes the foundation of this nonparametric estimation algorithm for the first time. This algorithm has been used in hundreds of peer-reviewed papers.

Pharmacometric observations can be described statistically by a mixture model. In this case, the probability of random variable arguments (the PK population model) of the pharmacokinetic compartmental model are described by a mixing distribution. The problem of estimating the mixing distribution from a set of pharmacometric observations can be stated as follows. Let $Y_{1},\ldots ,Y_{N}$ be a sequence of independent but not necessarily identically distributed random vectors constructed from one or more observations from each of *N* subjects in the population. Let $\theta_{1},\ldots ,\theta_{N}$ be a sequence of independent and identically distributed random vectors that represent unknown parameter values of *N* subjects. The θ belong to a compact subset Θ of Euclidean space with common but unknown distribution *F*. In other words, *F* is a distribution of the parameters in the population model. Θ represents the parameter space, and the dimension of this space is the number of parameters in the population model. The $\left\{\theta_{i}\right\}$ are not observed. It is assumed that the conditional densities $p(Y_{i}|\theta_{i})$ are known, for i=1,…,N. $p(Y_{i}|\theta_{i})$ represents the model of observations $y_{i}$ given parameter values $\theta_{i}$ including uncertainties of the measurement protocol. The mixing distribution of $Y_{i}$ with respect to *F* is given by $p\left(Y_{i}|F\right)=\int p\left(Y_{i}|\theta_{i}\right)dF\left(\theta_{i}\right)$. Because of independence of the $\left\{Y_{i}\right\}$, the mixing distribution of the $\left\{Y_{i}\right\}$ with respect to *F* is given by

(1) $$L\left(F\right)=p\left(Y_{1},...,Y_{N}|F\right)=\prod_{i=1}^{N}\int p\left(Y_{i}|\theta_{i}\right)dF\left(\theta_{i}\right)$$

The mixing distribution problem is to maximize the likelihood function L(F) with respect to all probability distributions *F* on Θ.

**Remark** **1.** *The distribution $F^{ML}$ that maximizes L(F) is a consistent estimator of the true mixing distribution; which means that $F^{ML}$ will converge to the true distribution if the number of subjects is large. This was proved originally by Kiefer and Wolfowitz in 1956 [4]. The consistency of $F^{ML}$ is especially important for our application to population pharmacokinetics where $F^{ML}$ is used as a prior distribution for Bayesian dosage regimen design [5].*

The algorithm described in this paper differs from most other published methods in a number of ways. Our algorithm allows for high dimensional parameter space Θ. Most published methods require the dimension of Θ to be small and many require the dimension of Θ to be 1, i.e., these methods required the number of parameters in the population model to be small. We have treated examples where the dimension of Θ is as high as 29, see Section 3.

Most published algorithms require the $\left\{Y_{i}\right\}$ to be identically distributed and assume that the population model $\left\{p\left(Y_{i}|\theta_{i}\right)\right\}$ is rather simple, such as $p(Y_{i}|\theta_{i})$ is a multivariate normal density with mean vector $\theta_{i}$ and covariance matrix Σ. Even if Σ is unknown and has to be estimated, the structure of this model is straightforward. However, the estimation of Σ has to be done carefully to avoid singularities, see Wang and Wang [6]. As will be described in Section 3, we allow $p(Y_{i}|\theta_{i})$ to be calculated from a system of nonlinear ordinary differential–algebraic equations.

We now describe the details of our algorithm. It was proved by Lindsay [7] and Mallet [8], under simple hypotheses on the population model $\left\{p\left(Y_{i}|\theta_{i}\right)\right\}$, that the global maximizer $F^{ML}$ of L(F) could be represented by a discrete distribution with support on at most *N* points, i.e., a distribution with nonzero probability located on at most *N* points.

**Remark** **2.** *One way to motivate this result by Lindsay and Mallet is as follows: Suppose by some lucky chance, we knew the exact parameters for each subject. How can we package this into a distribution? Answer: The “empirical distribution” of the exact parameters. That is the discrete distribution supported at the N exact parameters with equal weights. It turns out in this case that this empirical distribution is also the nonparametric maximum likelihood (NPML) distribution of the parameters. What is remarkable is that if we only have noisy measurements $\left(Y_{1},...,Y_{N}\right)$ of the N subjects only indirectly related to the subject parameters, the structure of the NPML is the same. A discrete distribution supported at N points. Of course, in this real case, the position and weights of the N support points are unknown. Finding these positions and weights is the subject of this paper.*

This result leads immediately to a finite dimensional optimization problem for $F^{ML}$, namely to maximize the likelihood function

(2) $$L\left(\lambda ,\phi \right)=\prod_{i=1}^{N}\sum_{k=1}^{K}\lambda_{k}p\left(Y_{i}|\phi_{k}\right)$$

with respect to the support points $\phi =(\phi_{1},\ldots ,\phi_{K})$ and weights $\lambda =(\lambda_{1},\ldots ,\lambda_{K})$ such that $\phi_{k}\in \Theta ,\lambda_{k}\geq 0$ for k=1,…,K, K≤N and $\sum_{k=1}^{K}\lambda_{k}=1$.

In our algorithm l(λ,ϕ)=logL(λ,ϕ) is maximized, so that

(3) $$l\left(\lambda ,\phi \right)=\sum_{i=1}^{N}\log\sum_{k=1}^{K}\lambda_{k}p\left(Y_{i}|\phi_{k}\right)$$

and the maximization problem becomes

(4) maximizel(λ,ϕ)

such that $\phi \in \Theta^{K}$, $\lambda =\left(\lambda_{1},...,\lambda_{K}\right)\in R_{+}^{K}$, K≤N and $\sum_{k=1}^{K}\lambda_{k}=1$.

Although the maximization problem in Equation (4) is finite dimensional, it is still high-dimensional. The dimension of the maximization problem in Equation (4) is N(dimΘ)+(N−1).

The optimization problem in Equation (4) is naturally divided into two problems:

Problem 1. Given a set of support points $\left\{\phi_{k}\right\}$, find the optimal weights $\left\{\lambda_{k}\right\}$.

Problem 2. Given the solution to Problem 1, find a better set of support points.

Problems 1 and 2 are solved cyclically until convergence, i.e., no significant improvement in l(λ,ϕ).

Problem 1 is a convex programming problem. In our algorithm, we solve this problem by the primal-dual interior-point (PDIP) method. This type of method is standard in convex optimization theory (see Boyd and Vandenberghe [9]). However, the exact implementation for a specific problem varies from problem to problem. The exact details of our implementation are described in the Appendix. See also Bell [10], Baek [11] and Yamada et al. [12]. Our PDIP implementation is fast and can easily handle thousands of variables.

Finding a better set of support points in Problem 2 is a more difficult problem. This location problem is a nonconvex global optimization problem with many local extrema and whose dimension is potentially N×dimΘ. The details of our algorithm, called the adaptive grid (AG) method, will be described in Section 2.3 and in Algorithm 1.
**Algorithm 1** Nonparametric adaptive grid (NPAG) algorithm. Input: $\left(Y,\phi^{0},a,b,\Delta_{D},\Delta_{L},\Delta_{F},\Delta_{e},\Delta_{\lambda }\right)$, a and b are the lists of lower and upper bounds, respectively, of Θ; $\Delta_{D}$ is the minimum distance allowable between points in the estimated $F^{ML}$. $\Delta_{x}$ see Section 2.7. Output: (ϕ,λ,l(λ,ϕ))1:**procedure**NPAG(Y, $\phi^{0},a,b,\Delta_{D}$)▹ Estimate $F^{ML}$ given Y2:      Initialization: $\phi =\phi^{0}$, $LogLike=-10^{30}$, $F_{0}=10^{30}$, $F_{1}=2*F_{0}$, eps=0.2, $\Delta_{e}=10^{-4}$, $\Delta_{F}=10^{-2}$, $\Delta_{L}=10^{-4}$, $\Delta_{\lambda }=10^{-3}$, n=03:      **while**
$eps\geq \Delta_{e}$ or $|F_{1}-F_{0}|\geq \Delta_{F}$
**do**4:        Calculate Ψ(ϕ)▹N×K matrix $\left\{p\left(Y_{i}|\phi_{k}\right)\right\}$5:        $\left[\hat{\lambda }\left(\phi \right),l\left(\hat{\lambda }\left(\phi \right),\phi \right)\right]⟵\mathrm{PDIP}\left(\Psi \left(\phi \right)\right)$▹ Appendix A6:        **if**
$\left(\mathrm{MAXCYCLES}==0\right)$
**then**7:           $F_{est}^{ML}⟵l\left(\hat{\lambda }\left(\phi \right),\phi \right)$8:           $\lambda ⟵\hat{\lambda }\left(\phi \right)$9:           **return**
$\left[\phi ,\lambda ,F_{est}^{ML}\right]$10:        **end if**11:        n⟵n+112:        $\phi^{c}⟵\mathrm{CONDENSE}\left(\phi ,\hat{\lambda }\left(\phi \right),\Delta_{\lambda }\right)$▹ Algorithm 313:        $\left[\hat{\lambda }\left(\phi^{c}\right),l\left(\hat{\lambda }\left(\phi^{c}\right),\phi^{c}\right)\right]⟵\mathrm{PDIP}\left(\Psi \left(\phi^{c}\right)\right)$▹$\mathrm{PDIP}\;\mathrm{returns}G^{n}$14:        $NewLogLike=l(\hat{\lambda }\left(\phi^{c}\right),\phi^{c})$15:        **if**
$\left(n>\mathrm{MAXCYCLES}\right)$
**then**16:           $F_{est}^{ML}⟵l\left(\hat{\lambda }\left(\phi^{c}\right),\phi^{c}\right)$17:           $\lambda ⟵\hat{\lambda }\left(\phi^{c}\right)$18:           **return**
$\left[\phi ,\lambda ,F_{est}^{ML}\right]$19:        **end if**20:        **if**
$\left|NewLogLike-LogLike\right|\leq \Delta_{L}$
**and**
$eps>\Delta_{e}$
**then**21:           eps=eps/2▹ Adjust precision22:        **end if**23:        **if**
$eps\leq \Delta_{e}$**then**▹ check EXIT conditions24:           $F_{1}=NewLogLike$25:           **if**
$|F_{1}-F_{0}|\leq \Delta_{F}$
**then**26:               $F_{est}^{ML}⟵F_{1}$27:               $\phi ⟵\phi^{c};\lambda ⟵\hat{\lambda }\left(\phi^{c}\right)$28:               **return**
$\left[\phi ,\lambda ,F_{est}^{ML}\right]$29:           **else**30:               $F_{0}=F_{1}$; eps=0.2▹ Reset Algorithm31:           **end if**32:        **end if**33:        $\phi ⟵\phi^{e}⟵\mathrm{EXPAND}\left(\phi^{c},eps,a,b,\Delta_{D}\right)$▹ Algorithm 234:        LogLike←NewLogLike35:    **end while**36:**end procedure**

Roughly speaking, Problems 1 and 2 are solved as follows. An initial large grid of possible support points is defined in the hypercube Θ. Problem 1 is solved on this large grid. After PDIP, most of the original grid points are removed due to near-zero weights, leaving a smaller high-probability grid. Problem 1 is then solved on this smaller grid. Then, the adaptive grid method for Problem 2 takes place. For each remaining grid point, up to 2×dimΘ new (daughter) support points are added. A daughter point outside the search space Θ or too close to a parent point is discarded. The new grid contains the current high-probability points plus the added daughter points. The algorithm is then ready for Problem 1 again. By construction, each iteration increases the value of l(λ,ϕ). This process continues until the function l(λ,ϕ) does not significantly change.

### 1.1. Comparable Methods

Because of space limitations, in this section, we only discuss NPML methods that optimize Equation (4), methods that treat multivariate distributions, and methods which allow general conditional probabilities $\left\{P\left(Y_{i},\theta_{i}\right)\right\}$. As explained in this paper, any such NPML algorithm has to address two problems: locations of support points and weights of support points. NPAG does locations by an adaptive grid method and weights by the primal-dual interior-point (PDIP) method. The algorithms discussed in this seection are summarized in Table 1.

The original methods of Lindsay [7] and Mallett [8] were based on algorithms of optimal design in the style of Fedorov [13]. In Schumitzky [14], an algorithm was proposed which did both locations and weights by the expectation maximization algorithm (EM). It was stable but also slow.

In Lesperance and Kalbfleisch [15], a new method was introduced which did weights by the dual method described in Section 5 of Lindsay [7] and locations by what they called the intra-simplex direction method (ISDM). Even though the Lesperance and Kalbfleisch paper was restricted to univariate distributions, the ISDM method has been generalized to the multivariate case. To briefly describe ISDM, let D(θ,F) be the directional derivative of logL(F) in the direction of the Dirac distribution $\delta_{\theta }$ supported at θ∈Θ. (This function is defined in Section 4 below). ISDM is an iterative algorithm. At stage *k*, let $F^{k}$ be the current estimate $F^{ML}$. Then, find all the local maxima of $D(\theta ,F^{k})$. These local maxima are added to the current set of support points and a new $F^{k+1}$ is calculated. If there are no new local maxima, then the algorithm is done.

In Pilla, Bartolucci, and Lindsay [16], another new method was developed where the locations were found by an initial fine grid. However, the weights were found by a dual version of the PDIP method.

In Savic, Kjellsson, and Karlsson [17], a nonparametric method (NONMEM-NP) was added to the popular NONMEM^®^ program. NONMEM-NP is a hybrid parametric–nonparametric approach The locations of support points were found by a parametric maximum likelihood algorithm. Then, the weights were found by maximizing Equation (4) relative to the newly found support points. NONMEM-NP can handle high-dimensional and complex multivariate distributions. An extension to NONMEM-NP was developed in Savic and Karlsson [18] where additional support points are added to the original set. A comparison between NONMEM-NP and NPAG is discussed in Leary [19].

In Wang and Wang [6], a new algorithm was developed for multivariate distributions. The locations were found by a combination of EM and a variant of ISDM. The weights were found by a family of quadratic programs. In [6], examples are performed for 8- and 13-dimensional mutivariate mixing distributions.

**Table 1:** Table of NPML Methods.

Author(s)/Reference/Date	Problem 1 Method	Problem 2 Method
Lindsay [7] (1983)	Convex Geometry	VDM
Mallet [8] (1986)	Optimal Design	VDM
Lesparance and Kalbfleisch [15] (1992)	Semi-Infinite Programming	ISDM
Savic, Kjellsson and Karlsson [17] (2009)	Parametric NONMEM	None
Pilla, Bartolucci and Lindsay [16] (2006)	Dual of PDIP	Adaptive Grid
Schumitzky [14] (1991)	EM	EM
X. Wang and Y. Wang [6] (2015)	Quadratic Programming	ISDM
NPAG [20] (2001)	PDIP	Adaptive Grid

Legend: VDM vertex direction method; EM expectation–maximization; ISDM intra-simplex direction method; NPML nonparametric maximum likelihood; PDIP primal-dual interior-point method.

Note: The quadratic programming algorithm (QP) of Wang and Wang [6] has an attractive feature. For a prescribed set of support points, QP finds the zero probabilities exactly. Thus, QP avoids the grid condensation step where support points from PDIP with sufficiently low probabilities are deleted. However, QP and PDIP are based on different numerical methods and a comparison of the efficiency of both algorithms has not been determined.

We finally mention that the NPML problem is a special case of a finite mixture model problem with unknown supports and weights. For a discussion of this approach, see Tatarinova and Schumitzky [21].

The algorithms which have shown by published examples to handle the highest dimensional multivariate problems are NONMEM-NP, Wang and Wang [6], and NPAG.

### 1.2. Benders Decomposition

For any set of grid points $\phi =(\phi_{1},...,\phi_{m})$ in $\Theta^{m}$, let $\lambda =\hat{\lambda }\left(\phi \right)$ be the corresponding set of optimal weights given by the PDIP method. Then, the function $F\left(\phi \right)=l(\hat{\lambda }\left(\phi \right),\phi )$ depends only on ϕ and can be maximized directly. For optimization methods, this technique is called Benders decomposition. The NPAG algorithm maximizes F(ϕ) by an adaptive search method. In a method proposed by James Burke, F(ϕ) is maximized by a Newton-type method. Since the function F(ϕ) is not necessarily differentiable, a relaxed Newton method must be used similar to what is described in the Appendix for the primal-dual algorithm. For details of Benders decomposition as applied to our problem, see Bell [10], Baek [11] and Jordan-Squire [22].

Founded on this prior work, the present study aimed to comprehensively describe, for the first time, the nonparametric adaptive grid algorithm (NPAG). This approach uses the exact log-likelihood to solve population modeling problems and does not make any assumptions about the shape of the PK parameter distributions. We illustrated the features and capabilities of this algorithm using a population PK modeling example. This algorithm presents the computational foundation of several hundred peer-reviewed papers, to date, and is ideally suited to optimize individual patient dosage regimens. The output of the NPAG algorithm becomes the input of the BestDose™ patient dosing software which is used at the bedside in real-time [23].

## 2. Materials and Methods

### 2.1. Pmetrics

The simulations and NPAG optimizations in this paper can be duplicated in R, using programs in the Pmetrics package [24]. R and Pmetrics are free software. R is available from many download sites. Pmetrics is available from lapk.org. NPAG is run using the NPrun() command in Pmetrics. Sample datasets and compartmental models are also available at lapk.org.

### 2.2. NPAG Subprograms

NPAG is a Fortran program consisting of a number of subroutines as described below. The main program performs the adaptive grid (AG) method (consisting of expansion and compression algorithms) and calls the primal-dual interior-point (PDIP) subprogram. The PDIP algorithm solves the maximization problem of Equation (4) for a fixed grid and is described precisely in the Appendix.

### 2.3. NPAG Implementation (NPAG—Algorithm 1)

For the purpose of this discussion, we can think of PDIP as a function $\hat{\lambda }$ from $\Theta^{m}$ into the set $S^{m}$ = $\left\{\lambda \in R_{+}^{m}:\sum_{k=1}^{m}\lambda_{k}=1\right\}$ defined as follows: If $\phi =(\phi_{1},...,\phi_{m})$ then $\hat{\lambda }\left(\phi \right)=\left({\hat{\lambda }}_{1},...,{\hat{\lambda }}_{m}\right)$ maximizes Equation (4) relative to the fixed set of grid points $\left(\phi_{1},...,\phi_{m}\right)$. In this case, we write *G* = $\left(\phi ,\hat{\lambda }\left(\phi \right)\right)$ and l(G) = $l(\phi ,\hat{\lambda }\left(\phi \right))$.

In NPAG, there are two types of grids: expanded and condensed. The expanded grids are the initial grid and the grids after grid expansion (Algorithm 2). The condensed grids are generated by grid condensation (Algorithm 3). Each cycle of NPAG begins with an expanded grid. The likelihood calculation is done on the condensed grids.

Now for the adaptive grid method. Assume that Θ is a bounded *Q*-dimensional hyper-rectangle. Initially, we let $\phi_{expanded}^{0}=\left(\phi_{1}^{0},...,\phi_{M}^{0}\right)$ be the set of *M* Faure grid points in Θ (see [25,26,27]). Alternatively, we could initially let $\phi_{expanded}^{0}$ be generated by a uniform distribution on Θ or by a prior run of the program.

Remark. The Faure grid points for a hyper-rectangle Θ are a low-discrepancy set which in some sense optimally and uniformly covers Θ. In our implementation of NPAG, the Faure point sets come in discrete sizes which nest with each other. (Allowable number of points equals 2129, 5003, 10,007, 20,011, 40,009, 80,021, and multiples of 80,021.) This nesting property is useful for checking the optimality of $F^{ML}$ (see Section 4). We have found that replacing the initial Faure set with a set generated by a uniform distribution on Θ increases the time to convergence but results in the same optimal distribution.

Now set $G_{expanded}^{0}$ = $\left(\phi^{0},\hat{\lambda }\left(\phi^{0}\right)\right)$. Our approach is to generate a sequence of solutions $G^{n}$ to Equation (4) of increasingly greater likelihood, where unless otherwise specified, $G^{n}$ refers to the condensed grid at the $n^{th}$ cycle of the algorithm. If $G^{n}$ has log-likelihood negligibly different than $G^{n-1}$, then $G^{n}$ is considered the optimal solution to Equation (4) and is relabeled $F^{ML}$. If not, then the process continues using the $\phi^{n}$ as the new seed. This loop is repeated until $F^{ML}$ is found.

The stopping conditions for NPAG are defined precisely in Algorithm 1. If the stopping conditions are not met prior to a set maximum number of iterations, the program will exit after writing the last calculated $G^{n}$ into a file.
**Algorithm 2** EXPAND. Input: ϕ = $\left(\phi_{1},\cdots ,\phi_{K}\right)$, $\Delta_{G}$, $\Theta =\left[a_{1},b_{1}\right]\times \left[a_{2},b_{2}\right]\times ...\times \left[a_{Q},b_{Q}\right]$, $a=[a_{1},\cdots ,a_{Q}]$, $b=[b_{1},\cdots ,b_{Q}]$, $\Delta_{D}$. Output: $\phi^{\prime }$=$\left(\phi_{1}^{\prime },\cdots ,\phi_{M}^{\prime }\right)$, where M≤K(1+2Q). Note: In this algorithm, ϕ = $\left(\phi_{1},\cdots ,\phi_{K}\right)$ is a Q×K matrix, with Q=dimΘ**function**Expand(ϕ, $\Delta_{G}$, a, b, $\Delta_{D}$)2:    Initialize: [Q,K]=size(ϕ), I=Q×Q Identity matrix, newϕ⟵ϕ    **for**
k=1,…,K
**do**▹K=number of of input support points4:        **for**
d=1,…,Q
**do**▹Q=dimΘ           $T\left(d\right)=\Delta_{G}\left(b\left(d\right)-a\left(d\right)\right)$6:           **if**
ϕ(d,k)+T(d)≤b(d)
**then**▹ Check upper boundary               $\phi^{+}=\phi \left(:,k\right)+T\left(d\right)I\left(:,d\right)$8:               $dist=10^{30}$           **end if**10:           **for**
$k_{in}=1:\mathrm{length}\left(\mathrm{new}\phi \right)$
**do**               $\mathrm{newdist}=\sum \mathrm{abs}\left(\phi^{+}-\mathrm{new}\phi \left(:,k_{in}\right)\right)./\left(b-a\right)$▹ x ./y donecomponent-wise12:               dist=min(dist,newdist)           **end for**14:           **if**
$dist\geq \Delta_{D}$
**then**▹ Check distance to new support point               $\mathrm{new}\phi ⟵\left[\mathrm{new}\phi ,\phi^{+}\right]$16:           **end if**           **if**
ϕ(d,k)−T(d)≥a(d)
**then**▹ Check lower boundary18:               $\phi^{-}=\phi \left(:,k\right)-T\left(d\right)I\left(:,d\right)$               $dist=10^{30}$20:           **end if**           **for**
$k_{in}=1:\mathrm{length}\left(\mathrm{new}\phi \left(1,:\right)\right)$
**do**22:               $\mathrm{newdist}=\sum (abs\left(\phi^{-}-\mathrm{new}\phi \left(:,k_{in}\right)\right)\;./\left(b-a\right))$▹ x./y donecomponent-wise               dist=min(dist,newdist)24:           **end for**           **if**
$dist\geq \Delta_{D}$
**then**▹ Check distance to new support point26:               $\mathrm{new}\phi ⟵[\mathrm{new}\phi ,\phi^{-}]$           **end if**28:        **end for**    **end for**30:    ϕ⟵newϕ**end function****Algorithm 3** Condense algorithm. Input: $\left(\phi ,\lambda ,\Delta_{\lambda }\right)$, Output: $\phi^{c}$ Note: $\phi^{c}$ is considered a subset of ϕ
**function**Condense($\phi ,\lambda ,\Delta_{\lambda }$)     **ind** = **find** ( $\lambda >\left(\max\lambda \right)\Delta_{\lambda }$ )▹ Inequality and max are performed component-wise     $\phi^{c}=\phi \left(:,\mathrm{ind}\right)$     return $\phi^{c}$**end function**

### 2.4. Grid Expansion (EXPAND—Algorithm 2)

The crux of the adaptive grid method is how to go from $G^{0}$ to $G^{1}$ or, more generally, from $G^{n}$ to $G^{n+1}$. The details of doing this are now explained roughly below and precisely in Algorithm 1.

Let *Q* be the dimension of Θ. Suppose at stage *n* we have a grid of high-probability support points $\phi^{n}$. We then add 2Q daughter points for each support point $\phi_{k}\in \phi^{n}$. The daughter points are the vertices of a small hyper-rectangle centered at each $\phi_{k}$ with size proportional to the original size of the hyper-rectangle defining Θ. The size of this small hyper rectangle decreases as the accuracy of the estimates increases. (See Algorithm 2).

Let $\phi_{expanded}^{n+1}$ = $\phi^{n}\cup \mathrm{Daughter}-\mathrm{Points}$. Then the PDIP subprogram is applied to $\phi_{expanded}^{n+1}$ resulting in the new solution set $G_{expanded}^{n+1}$ = $\left(\phi_{expanded}^{n+1},\hat{\lambda }\left(\phi_{expanded}^{n+1}\right)\right)$ (see Algorithm 1). The solution set $G_{expanded}^{n+1}$ is now ready for grid condensation.

### 2.5. Grid Condensation (CONDENSE—Algorithm 3)

The above solution set $G_{expanded}^{n+1}$ may have many support points with low probability. We remove all support points which have corresponding probability less than $\left(\max\lambda \right)\Delta_{\lambda }$, where λ is the vector of current probabilities and the default for $\Delta_{\lambda }$ is $10^{-3}$. (Note that the remaining probabilities are not normalized at this point.) The probabilities of the remaining support points are normalized by a second call to the PDIP subprogram. This second call to PDIP is fast. The likelihood associated with these remaining support points and normalized probabilities is then used to update the program control parameters and check for convergence (Algorithm 1 and Section 2.7). If convergence is attained, then the output of this second call to PDIP provides the support points and probabilities of the final solution. If convergence is not attained, then the remaining support points are sent to the grid expansion subprogram (Algorithm 2), initializing the next cycle.

At the end of the program, the output of this second call to PDIP provides the location and weights of the final solution.

### 2.6. PDIP Subprogram—See Appendix A

The PDIP subprogram finds the optimal solution to Equation (4) with respect to λ for fixed ϕ. PDIP employs a primal-dual interior-point method that uses a relaxed Newton method to solve the corresponding Karush–Kuhn–Tucker equations. (See Equations (14)–(17) of Appendix A).

For any Y=($Y_{1},...,Y_{N}$) and any ϕ=($\phi_{1},...,\phi_{K})\in \Theta^{K}$, the input to the PDIP subprogram is the N×K matrix $\left\{p(Y_{i}|\phi_{k})\right\}$. The output consists of the optimal weights $\hat{\lambda }\left(\phi \right)$ and the corresponding log-likelihood $l(\hat{\lambda }\left(\phi \right),\phi )$. An in-depth description of the PDIP algorithm and its implementation is presented in Appendix A. See also [10,11,12].

### 2.7. NPAG Stopping Conditions

As explained above, a potential solution to $F^{ML}$ is not accepted as a global optimum until successive sequences of $G^{n}$ produce final distributions evaluating to sufficiently close log-likelihood. The various upper and lower bounds Δ for NPAG control and stopping conditions are defined below and are used in Algorithms 1–3.

Δ_L_ Primary upper bound on the allowable difference between two successive estimated log-likelihoods; the default initialization is $10^{-4}$.
Δ_F_ Secondary upper bound on the allowable difference between two successive estimated log-likelihoods of potential $F^{ML}$; the default initialization is $10^{-2}$.
Δ_e_ Sets an upper bound on the accuracy variable eps of Algorithm 1. The default initialization for $\Delta_{e}$ is $10^{-4}$. The default initialization for eps is 0.2 and is stepped down until $eps\leq \Delta_{e}$ $\Delta_{F}$ and $\Delta_{e}$ define the two stopping conditions for Algorithm 1.
Δ_D_ Sets a lower bound on how close two support points can get; the default initialization is $10^{-4}$.
Δ_λ_ Sets a lower bound factor on the probabilities of the weights λ; the default initialization is $10^{-3}$.

### 2.8. Calculation of $p(Y_{i}|\phi_{k})$

Given observations $Y_{i}$, i=1,...,N and grid points $\phi_{k}$, k=1,...,K, the PDIP subprogram only depends on the N×K matrix $\left\{p(Y_{i}|\phi_{k})\right\}$. NPAG can be used for any problem once this matrix is defined. However, the default setting of NPAG is for the problem of population pharmacokinetics. For a good background of population pharmacokinetics, see Davidian and Giltinan [28,29].

In population pharmacokinetics, generally $Y_{i}=\left(y_{i,1},...,y_{i,M}\right)$ is a matrix of vector observations for the *i*th subject. Since NPAG allows multiple outputs, each $y_{i,m}$ is itself a *q*-dimensional vector $y_{i,m}$ =$\left(y_{i,m,1},\cdots ,y_{i,m,q}\right)$. The observations $y_{i,m,j}$, are then typically given by a regression equation of the form:

(5) $$\begin{array}{cc} y_{i,m,j} & =f_{i,m,j}\left(\theta_{i}\right)+\nu_{i,m,j},\;j=1,\cdots ,q\\ \nu_{i,m,j} & ∼N(0,{\left(\sigma_{i,m,j}\left(\theta_{i}\right)\right)}^{2})\\ \theta_{i} & \mathrm{are}\;\mathrm{unobserved}\;\mathrm{parameters}\;\mathrm{specific}\;\mathrm{for}\;Y_{i} \end{array}$$

In the above Equation (5), $f_{i,m,j}$ is a known nonlinear function depending on the model structure, the dosage regimen, the sampling schedule, all covariates and of course the subject-specific parameter vector $\theta_{i}$. Except for simple models, $f_{i,m,j}$ requires the solution of (possibly nonlinear) ordinary differential equations.

In the current implementation of NPAG, it is assumed that the $\left(y_{i,1},...,y_{i,M}\right)$ are independent. Then

(6) $$p\left(Y_{i}|\phi_{k}\right)=\frac{\exp\left(-\frac{1}{2}\sum_{m=1}^{M}\left(y_{i,m}-f_{i,m}\left(\phi_{k}\right)\right)\Sigma_{i,m}^{-1}\left(\phi_{k}\right){\left(Y_{i,m}-f_{i,m}\left(\phi_{k}\right)\right)}^{T}\right)}{\prod_{m=1}^{M}\sqrt{{\left(2\pi \right)}^{q}\det\Sigma_{i,m}\left(\phi_{k}\right)}}$$

where $f_{i,m}=\left(f_{i,m,1},...,f_{i,m,q}\right)$ and $\Sigma_{i,m}$ = $diag(\sigma_{i,m,1}^{2},...,\sigma_{i,m,q}^{2})$. For the purposes of matrix multiplication in Equation (6),we think of $y_{i,m}$ and $f_{i,m}$ as *q*-dimensional row vectors.

To complete the description of Equation (6) we need to model the standard deviation terms $\sigma_{i,m,j}$ of the assay noise. In our implementation of NPAG, four different models are allowed. Let

(7) $$\alpha_{i,m,j}\left(\phi_{k}\right)=c_{0}+c_{1}f_{i,m,j}\left(\phi_{k}\right)+c_{2}f_{i,m,j}^{2}\left(\phi_{k}\right)+c_{3}f_{i,m,j}^{3}\left(\phi_{k}\right)$$

and set

(8) $$\begin{array}{cc} \sigma_{i,m,j} & =\left\{\begin{array}{cc} \alpha_{i,m,j} & \mathrm{assay}\;\mathrm{error}\;\mathrm{polynomial}\;\mathrm{only}\\ \gamma \alpha_{i,m,j} & \mathrm{multiplicative}\;\mathrm{error}\\ \sqrt{\alpha_{i,m,j}^{2}+\gamma^{2}} & \mathrm{additive}\;\mathrm{error}\\ \gamma & \mathrm{constant}\;\mathrm{level}\;\mathrm{of}\;\mathrm{error} \end{array}\right. \end{array}$$

The parameter γ in Equation (8) is a variance factor. Artificially increasing the variance during the first several cycles of NPAG increases the likelihood for each ϕ, allowing the algorithm to use these cycles to find a better initial state from which to begin optimization. NPAG also has an option to “optimize” γ. This changes NPAG from a nonparametric method to a “semiparametric” method and will not be discussed here. The interested reader can consult [12].

Next, if $c_{0}=0$ in Equation (7), then $\alpha_{i,m,j}$ can become small for certain values of ϕ that in early iterations can be far from optimal. This, in turn, causes numerical problems as the likelihood is infinite if $\sigma_{i,m,j}=0$. One way to avoid this problem is to take $\sigma_{i,m,j}=constant$. Another way would be to assume that $\alpha_{i,m,j}$ is known and is given by

(9) $$\alpha_{i,m,j}=c_{0}+c_{1}y_{i,m,j}+c_{2}y_{i,m,j}^{2}+c_{3}y_{i,m,j}^{3}$$

That is, to approximate σ by using a polynomial of the observed values rather than model predicted values. In our experience with NPAG, the approximation of Equation (9) is useful for ensuring computational stability (especially during the early cycles of the algorithm). However, from a theoretical perspective, this change violates the conditions of maximum likelihood and will not be discussed here. Again, the interested reader can consult [12].

### 2.9. Convergence

For a given initial grid $\phi^{0}$, the NPAG algorithm is only guaranteed to find a local maximum of L(F). More precisely, if $\phi^{*}$ is the final grid of NPAG starting from $\phi^{0}$, then $\hat{\lambda }\left(\phi^{*}\right)$ is a global maximum on $\phi^{*}$ but the support points $\phi^{*}$ may be only a local maximum.

Global convergence of a nonparameteric maximum likelihood method for estimation of a multivariate mixing distribution is difficult. For one-dimensional distributions the problem is straightforward. The idea of proof goes back to at least Fedorov [13] in 1972, which involves the use of directional derivatives.

Let *F* be any distribution on Θ. Then, the directional derivative of logL(F) in the direction of the Dirac distribution $\delta_{\theta }$ supported at θ is defined by

D(θ,F) = [$\sum_{i=1}^{N}P\left(Y_{i}|\theta \right)/P\left(Y_{i}|F\right)]-N$, θ∈Θ, where $p\left(Y_{i}|F\right)=\int p\left(Y_{i}|\theta \right)dF\left(\theta \right)$. Let $F_{k}$ be the current NPML estimate at iteration *k*. The Fedorov method involves maximizing $D(\theta ,F_{k})$ for θ∈Θ, at every iteration. Then, the point at which the maximum occurs is added in an optimal way to $F_{k}$ to give $F_{k+1}$. Under the assumptions of regularity, Fedorov shows that $L(F_{k})$ converges to $L(F^{ML})$, see Fedorov [13], (Theorem 2.5.3). Many improvements to this method have been made. In Lesperance and Kalbfleisch [15] and Wang and Wang [6], instead of just adding the point at which $D(\theta ,F_{k})$ occurs, all the points where local maxima occur are added in an optimal way. Again, under the assumptions of regularity, convergence as above is proved. In one dimension, these methods are efficient. In higher dimensions, these methods are not computationally practical.

We now suggest a method to check whether the final distribution of NPAG is globally optimal and, if not optimal, how close it is to the optimal. It also involves the use of the directional derivative D(θ,F), but only at the last iteration of NPAG. Now define

$$D\left(F\right)=\max_{\theta \in \Theta }D\left(\theta ,F\right)$$

Note that the max in the above expression is only over Θ and not over $\Theta^{N}$. It is proved in Lindsay [7] that $F^{*}$ is a global maximum of L(F), i.e., $F^{*}$=$F^{ML}$, if and only if $D(F^{*})=0$. Even if $D(F^{*})\neq 0$, it is useful to make this computation as it is also proved in Lindsay [7] that $L\left(F^{ML}\right)-L\left(F^{*}\right)\leq D\left(F^{*}\right)$, so this last expression gives an estimate of the accuracy of the final NPAG result.

Now, even though we said above it is not practical to calculate D(F) at every iteration of an algorithm, we are just suggesting to make this calculation at the end of the algorithm. This calculation can be performed by a deterministic or stochastic optimization algorithm.

## 3. Examples

First of all, the NPAG program has been used successfully in high-dimensional and very complex pharmacokinetic–pharmacodynamic models. In Ramos-Martin et al. [30], the NPAG program was used for a population model of the pharmacodynamics of vancomycin for coagulase-negative staphylococci (CoNS) infection in neonates. Vancomycin is an antibiotic used to treat a number of serious bacterial infections. CoNS are the most commonly isolated pathogens in the neonatal intensive care unit. This model had 7 nonlinear differential equations and 11 random parameters. The population was a combination of 300 experimental and animal subjects. In Drusano et al. [31], the NPAG program was used for a population model of two drugs for the treatment of tuberculosis. This model had 5 nonlinear differential equations, 3 nonlinear algebraic equations, 1671 observations from 6 outputs and 29 random parameters. In the algebraic equations, the state variables were only defined implicitly and had to be solved for by an iterative method.

The above two examples are too complex to use for simulation purposes. Consequently, we present here a simpler model which has an analytic solution and which can be checked by other algorithms. Nevertheless, the estimation of parameters in this model is not trivial. We consider a three-compartment PK model with a continuous IV infusion into the central compartment and a bolus input into the absorption compartment. The individual subject model is described by the following differential equations:

$$\begin{array}{cc} \frac{dx_{1}}{dt} & =-K_{a}x_{1},\;x_{1}\left(t\right)=\left\{\begin{array}{cc} 0 & \mathrm{for}\;0\leq t<5\\ b & \mathrm{if}\;t=5 \end{array}\right.\\ \frac{dx_{2}}{dt} & =K_{a}x_{1}-\left(K_{e}+K_{cp}\right)x_{2}+K_{pc}x_{3}+r\left(t\right),\;x_{2}\left(0\right)=0\\ \frac{dx_{3}}{dt} & =K_{cp}x_{2}-K_{pc}x_{3},\;x_{3}\left(0\right)=0 \end{array}$$

and output equation

$$y_{1}\left(t\right)=x_{2}\left(t\right)/V_{c}+w\left(t\right),w\left(t\right)∼N\left(0,\sigma^{2}\right),\;\sigma =5.5\;$$

**Figure A1:**
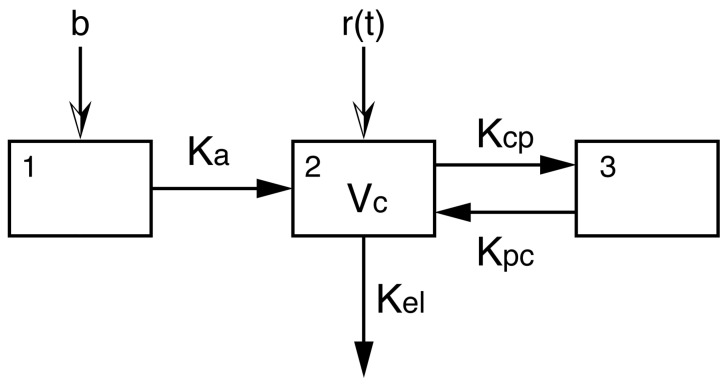
Model.

The inputs are a bolus b=2000 mg at t=5 Hr and a continuous infusion r(t)=500 Hr${}^{-1}$, for 0<t<16 Hr. This model has 5 random parameters (*V*, $K_{a}$, $K_{e}$, $K_{cp}$, $K_{pc}$). A diagram of this model is given in Figure A1. It is known that this model is structurally identifiable, see Godfrey [32]. However, we have found that for a continuous IV infusion, the parameters $K_{cp}$ and $K_{pc}$ can be difficult to estimate in a noisy environment.

The details of the simulation are as follows. There were 300 simulated subjects. The random variables $\left(V,\;K_{a},\;K_{cp},\;K_{pc}\right)$ were independently simulated from normal distributions with means respectively equal to (1.2,0.8,2.0,0.2) and standard deviations equal to 25% coefficient of variation.

The random variable $K_{e}$ was independently simulated from a bimodal mixture of two normal distributions with means respectively equal to 0.5 and 1.5, with standard deviations equal to 10% coefficient of variation, and with weights equal to 0.2 and 0.8. This distribution would apply to an elimination rate constant with a bimodal distribution where 80% of the subjects have a mean of 1.5, and only 20% have a mean of 0.5. The power of the nonparametric method allows the detection of the 20% group.

Eleven observations were taken at times t=0.25,1.0,4.98,5.25,5.5,6.0,7.0,8.5,10.0, 13.0,16.0.

**Figure A2:**
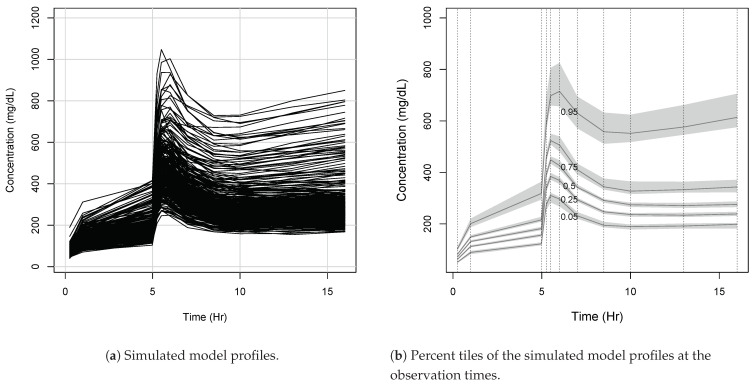
The 300 simulated observed profiles, frame a, are generated in Pmetrics using the function SIMrun(). The 0.05, 0.25, 0.5, 0.75, and 0.95 percent tiles of the profiles are plotted in frame b, with the $CI_{95\%}$ in grey. The observation times are marked by a vertical dotted line in frame b. Other details are in the text. Of note: inspection of these profiles does not suggest a bimodal elimination parameter.

These sampling times were chosen in an ad hoc fashion and are not to be considered optimal. In Figure A2, we show the profiles of the 300 noisy model outputs $y_{1}$. These profiles are plotted as piecewise linear functions with nodes at the observation times.

The initial Faure set had 80,321 support points dispersed in the volume

$$\left(K_{a},V,K_{e},K_{cp},K_{pc}\right)\in \left[(0.01,2.0),(0.01,2.5),(0.0001,2.0),(0.0,4.0),(0.0001,2.0)\right]$$

The assay error (Equation (8)) is not always known. An approximate assay error polynomial can be inferred from literature and Pmetrics includes a routine to estimate the assay error polynomial from the data. Another approach to analyzing data with unknown measurement error is to run successive NPAG optimizations using decreasing error magnitude for each new run. An advantage of this approach is that model development is faster. The first cycle of NPAG, which begins with a relatively large measurement error, can be initialized using a relatively small number of support points. Each NPAG solution is used as a prior to skip the first (and most computationally burdensome) step in the next NPAG run. We demonstrate this approach can converge to the correct solution on this simulated data.

Convergence for this problem was accomplished after applying NPAG four times. For the first application, $\sigma =0.025Y_{simulated}$. The output distribution of this first application was used as a prior to start NPAG again, this time with $\sigma =7.96+0.0125Y_{simulated}$. The output of this second application was used as a prior to start a third NPAG run, this time with $\sigma =7.96+0.0065Y_{simulated}$. Finally, the output density of this third run was used as a prior to run NPAG a fourth time, this time with σ=5.5, the same as for the simulation. The step down in assumed observation error happened at convergence for each previous error levels: at cycles 4513, 5972, and 6791. Final convergence happened at cycle 8012. There are 284 support points in the final density.

**Figure A3:**
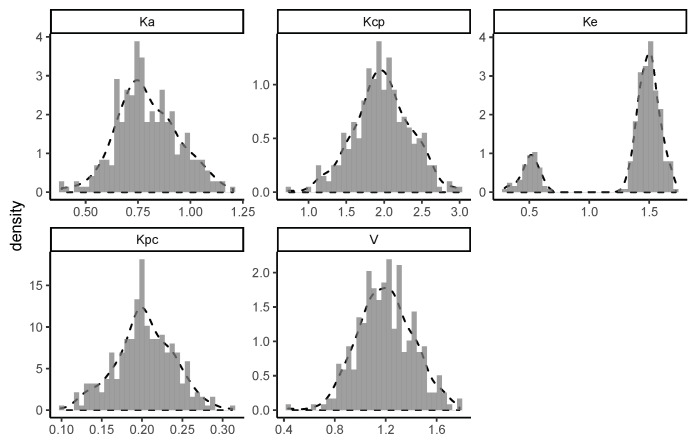
Kernel densities of simulated PK parameters. All *K* are in $Hr^{-1}$, Volume is in dL. Kernel density (dashed line) of the distribution is over-plotted on the histogram (grey), which are normalized to the total observations. Simulated support points are generated in Pmetrics using the function SIMrun(). Details are in the text. Kernel densities are calculated in R using the (S3) generic function density().

**Figure A4:**
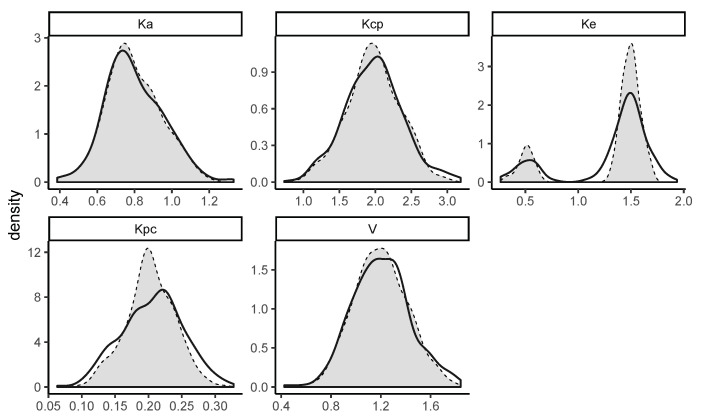
Kernel densities of NPAG estimated PK parameters (black line) vs. Simulated r.v.s (dashed with grey fill). *K* are in $Hr^{-1}$, volume is in dL. NPAG estimation is calculated using the NPrun() function in Pmetrics. Similarity of these two distributions is verified in R using the function mtsknn.eq(). See text for further detail.

The simulated and estimated marginal distributions are shown in Figure A3 and Figure A4. It is seen that the estimated marginal distributions are similar to the simulated histograms. In particular, the bimodal shape of $K_{e}$ was uncovered. Similarity is tested using the R routine mtsknn.eq(..., k = 3), which returns a pval=0.5809. mtsknn.eq applies a *K*—nearest neighbors approach to estimate the probability that two non-parametric distributions arise from the same distribution.

NPAG is designed to estimate the whole joint distribution of the parameters. As mentioned earlier, the estimate $F^{ML}$ is especially important for our application to population pharmacokinetics where $F^{ML}$ is used as a prior distribution for Bayesian dosage regimen design. However, $F^{ML}$ is a consistent estimator of the true mixing distribution and consequently, the moments of $F^{ML}$ should be consitent estimators of the true moments. Means and variances of parameter estimates for $F^{ML}$ can be easily obtained by integrating the corresponding marginal distributions. So as a check of this fact, in Table A1, the comparisons of estimated versus simulated means and variances are shown. Again, results are quite accurate (see Table A1 and Table A2).

**Figure A5:**
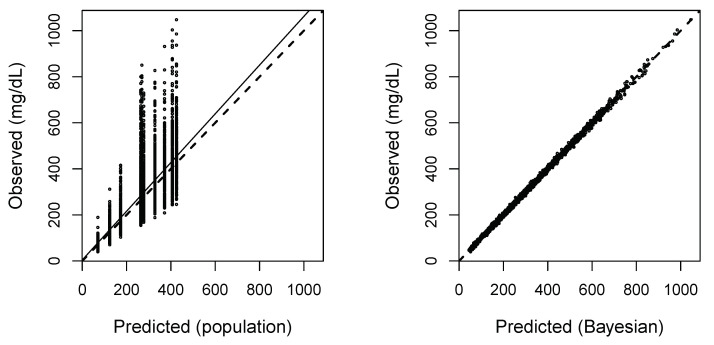
Predicted vs. Observed. Population Fit: $r^{2}=0.569$, intercept $=3.8(CI_{95\%}-5.85,13.5)$, slope $=1.12(CI_{95\%}1.08,1.15)$, bias =−6.45, imprecision =366. Individual Bayesian Posterior Fit: $r^{2}=0.999$, intercept $=0.168(CI_{95\%}-0.267,0.603)$, slope $=0.998(CI_{95\%}0.997,1)$, bias =0.0612, imprecision =1.15.

Finally, in Figure A5 we include a graph of Predicted versus Observed values which shows the all around good fit of the data. The predicted (right panel: Predicted Bayesian) values are gotten as follows: For each subject, the Bayesian mean estimate of the parameters are found using the final NPAG distribution as a prior and that subject’s observations. Then, based on these parameter means, the subject’s concentration profile is calculated. The predicted (left panel: Predicted Population) is the weighted average of the evaluation of all support points for subject model.

## 4. Conclusions

We have provided the first comprehensive description of the NPAG algorithm for estimating multivariate mixing distributions. This algorithm can describe between subject variability without assuming any shape for the distribution of PK parameters and is excellently suited for optimizing patient dosing [33,34,35]; NPAG is based on an iterative algorithm employing the Primal-Dual Interior-Point method and an Adaptive Grid method. This approach can handle pharmacokinetic, pharmacodynamic and other models with a high number of estimated model parameters and is based on the exact log-likelihood. A detailed description of NPAG is provided along with an application for a common two-compartment PK models. Finally, the NPAG algorithm is arguably the most efficiently parallelizable population modeling algorithm, since it can parallelize over both subjects and support points. This allows one to readily implement this algorithm on supercomputers as our laboratory has done on research projects. In addition to population pharmacokinetics, this research also applies to empirical Bayes estimation, see Koenker and Mizera [36] and to many other areas of applied mathematics, see Banks et al. [37]. Overall, the NPAG algorithm provides an important addition to the pharmacometric toolbox for drug development and optimal patient dosing at the interface of applied mathematics, biomedical scientists, and clinicians.

## 5. Supplementary Material

### 5.1. Recent References Using Npag

As a means for readers to more quickly assess the applicability of NPAG in a wide variety of pharmacometric studies, we refer here to the first 10 references on a recent PubMed search for papers that use NPAG. Note that all population PK studies using Pmetrics employ NPAG.

1. Pharmacodynamics of Posaconazole in Experimental Invasive Pulmonary Aspergillosis: Utility of Serum Galactomannan as a Dynamic Endpoint of Antifungal Efficacy. Gastine S, Hope W, Hempel G, Petraitiene R, Petraitis V, Mickiene D, Bacher J, Walsh TJ, Groll AH. Antimicrob Agents Chemother. 2020 Nov 9:AAC.01574-20. doi: 10.1128/AAC.01574-20. Online ahead of print. PMID: 33168606
2. Cerebrospinal fluid penetration of ceftolozane/tazobactam in critically ill patients with an indwelling external ventricular drain. Sime FB, Lassig-Smith M, Starr T, Stuart J, Pandey S, Parker SL, Wallis SC, Lipman J, Roberts JA. Antimicrob Agents Chemother. 2020 Oct 19:AAC.01698-20. doi: 10.1128/AAC.01698-20. Online ahead of print. PMID: 33077655
3. Population Pharmacokinetics of Continuous-Infusion Meropenem in Febrile Neutropenic Patients with Hematologic Malignancies: Dosing Strategies for Optimizing Empirical Treatment against Enterobacterales and P. aeruginosa. Cojutti PG, Candoni A, Lazzarotto D, Filì C, Zannier M, Fanin R, Pea F. Pharmaceutics. 2020 Aug 19;12(9):785. doi: 10.3390/pharmaceutics12090785. PMID: 32825109 Free PMC article.
4. Caspofungin Weight-Based Dosing Supported by a Population Pharmacokinetic Model in Critically Ill Patients. Märtson AG, van der Elst KCM, Veringa A, Zijlstra JG, Beishuizen A, van der Werf TS, Kosterink JGW, Neely M, Alffenaar JW. Antimicrob Agents Chemother. 2020 Aug 20;64(9):e00905-20. doi: 10.1128/AAC.00905-20. Print 2020 Aug 20. PMID: 32660990 Free PMC article.
5. Ethionamide Population Pharmacokinetic Model and Target Attainment in Multidrug-Resistant Tuberculosis. Al-Shaer MH, Märtson AG, Alghamdi WA, Alsultan A, An G, Ahmed S, Alkabab Y, Banu S, Houpt ER, Ashkin D, Griffith DE, Cegielski JP, Heysell SK, Peloquin CA. Antimicrob Agents Chemother. 2020 Aug 20;64(9):e00713-20. doi: 10.1128/AAC.00713-20. Print 2020 Aug 20. PMID: 32631828
6. Development and validation of a dosing nomogram for amoxicillin in infective endocarditis. Rambaud A, Gaborit BJ, Deschanvres C, Le Turnier P, Lecomte R, Asseray-Madani N, Leroy AG, Deslandes G, Dailly É, Jolliet P, Boutoille D, Bellouard R, Gregoire M; Nantes Anti-Microbial Agents PK/PD (NAMAP) study group. J Antimicrob Chemother. 2020 Oct 1;75(10):2941-2950. doi: 10.1093/jac/dkaa232. PMID: 32601687
7. Population Pharmacokinetics and Target Attainment of Cefepime in Critically Ill Patients and Guidance for Initial Dosing. Al-Shaer MH, Neely MN, Liu J, Cherabuddi K, Venugopalan V, Rhodes NJ, Klinker K, Scheetz MH, Peloquin CA. Antimicrob Agents Chemother. 2020 Aug 20;64(9):e00745-20. doi: 10.1128/AAC.00745-20. Print 2020 Aug 20. PMID: 32601155
8. Baclofen self-poisoning: Is renal replacement therapy efficient in patient with normal kidney function? Brunet M, Léger M, Billat PA, Lelièvre B, Lerolle N, Boels D, Le Roux G. Anaesth Crit Care Pain Med. 2020 Oct 14:S2352-5568(20)30230-7. doi: 10.1016/j.accpm.2020.07.021. Online ahead of print. PMID: 33068797
9. Ceftriaxone dosing in patients admitted from the emergency department with sepsis. Heffernan AJ, Curran RA, Denny KJ, Sime FB, Stanford CL, McWhinney B, Ungerer J, Roberts JA, Lipman J. Eur J Clin Pharmacol. 2020 Sep 24. doi: 10.1007/s00228-020-03001-z. Online ahead of print. PMID: 32974748
10. Population Pharmacokinetic Models of Anti-Tuberculosis Drugs in Patients: a Systematic Critical Review. Otalvaro JD, Hernandez B E AM, Rodriguez CA, Zuluaga AF. Ther Drug Monit. 2020 Sep 18. doi: 10.1097/FTD.0000000000000803. Online ahead of print. PMID: 32956238

### 5.2. Recent Studies to Which NPAG Can Be Applied

The references below use excellent parametric methods. All of these methods have the same mathematical structure as our nonparametric NPAG. The main difference is that in the parametric methods, it is assumed that the population distribution is multivariate normal with unknown mean vector and unknown covariance matrix. In NPAG, we do not make any parametric assumptions about the population distribution, as discussed in our paper. Otherwise, the population analysis problem is the same. Bulitta et al. [38] and Shah et al. [39] use the method S-ADAPT. S-ADAPT is based on the ADAPT package developed by D’Argenio, Wang and Schumitzky. Ishihara et al. [40] and Soraluce et al. [41] use the industry standard parametric version of NONMEM. Allard et al. [42] uses the stochastic approximation expectation maximization method (SAEM). NPAG can run any problem that is run by any of these programs.

### 5.3. Comparison of NPAG to Classical Population Analysis Programs

Several studies have compared NPAG to a parametric algorithm. Bustad et al. [43] compared the statistical consistency and efficiency of ITS and two older NONMEM^®^ routines, FO and FOCE to that of NPEM and NPAG on simulated datasets; the nonparametric methods were more consistent and efficient. Prémaud et al. [44] also compared NPAG to NONMEM^®^ FOCE, but the two algorithms converged to distinctly different results. NPAG converged to an estimator with better predictive performance allowing for its use in therapeutic drug monitoring. More recently, de Velde et al. [45] compared NONMEM^®^ FOCE-I to NPAG, with both methods converging to similar parameter estimates. In each of the above studies, NPAG converged to a distribution with greater variance.

## Data Availability

Data is available on request from contact@lapk.org.

## Abbreviations

The following abbreviations are used in this manuscript:

AG	Adaptive grid
CoNS	Coagulase-negative Staphylococci
EM	Expectation–maximization algorithm
ISDM	Intra-simplex direction method
NPAG	Nonparametric adaptive grid algorithm
NPML	Nonparametric maximum likelihood
PDIP	Primal-dual interior-point method
QP	Quadratic programming

## Appendix A. A Primal-Dual Interior-Point Algorithm (PDIP)

To make this paper self-contained, we outline here the PDIP algorithm which was written by James Burke. This algorithm is a FORTRAN subroutine of NPAG. The description below is based on the Matlab and C++ codes found in Bradley Bell’s website, see [10]. Definition of general terms and theorems can be found in Boyd and Vandenberghe [9].

### Appendix A.1. Duality Theory and the Basic Problem

Given a set of support points $\left\{\phi_{k}\right\}$, the problem of finding the optimal weights $\left\{\lambda_{k}\right\}$ in Equation (4) can be posed as the following optimization problem

$$P\;\min\Phi \left(\Psi \lambda \right)\;s.t.\;0\leq \lambda ,\;e^{⊺}\lambda =1,$$

where $\Psi \in R^{n\times m}$ is the matrix whose (i,j) entry is $p(y_{i}|\phi_{j})$ and where in general, the function $\Phi :R^{k}\mapsto R\cup \left\{+\infty \right\}$ is given by

(A1) $$\Phi \left(z\right)=\left\{\begin{array}{cc} -\sum_{i=1}^{k}\log z_{i} & ,0<z,\mathrm{and}\\ +\infty & ,\mathrm{otherwise}. \end{array}\right.$$

The symbol e is always to be interpreted as the vector of all ones of the appropriate dimension.

The problem P is a convex programming problem since the objective function Φ is convex and the constraining region is a convex set. The Fenchel–Rockafellar dual of the convex program P is the problem

$$D\;\min_{}\Phi \left(\omega \right)\;s.t.\;L^{⊺}\omega \leq me.$$

From Boyd, we obtain the following Karush–Kuhn–Tucker (KKT) equations relating the solutions to the problem P and D.

(A2) $$\begin{array}{cc} me & =\Psi^{⊺}w+y \end{array}$$

(A3) $$\begin{array}{cc} e & =W\Psi \lambda \end{array}$$

(A4) $$\begin{array}{cc} 0 & =\Lambda Ye \end{array}$$

where for any vector x, we define X to be the diagonal matrix having x along the diagonal.

### Appendix A.2. An Interior-Point Path-Following Algorithm

The relaxed KKT is given by

(A5) $$\begin{array}{cc} me & =\Psi^{⊺}w+y \end{array}$$

(A6) $$\begin{array}{cc} e & =W\Psi \lambda \end{array}$$

(A7) $$\begin{array}{cc} \mu e & =\Lambda Ye \end{array}$$

(A8) $$\begin{array}{cc} \; & 0\leq \lambda ,\;0\leq w,\;0\leq y, \end{array}$$

for μ>0. (μ is the relaxation parameter.) A damped Newton’s method is used to solve the above system.

Consider the function $F:\;R^{2m+n}\mapsto R^{2m+n}$ given by

$$F\left(\lambda ,w,y\right)=\left[\begin{array}{c} \Psi^{⊺}w+y\\ W\Psi \lambda \\ \Lambda Ye \end{array}\right].$$

A triple (λ,w,y) solves Equations (A5) to () if and only if

(A9) $$F\left(\lambda ,w,y\right)=\left(\begin{array}{c} me\\ e\\ \mu e \end{array}\right)$$

and 0≤λ, 0≤ω, and 0≤y. Path-following algorithms attempt to solve (A9) by applying Newton’s method for progressively smaller values of the relaxation parameter μ. We first need the derivative of *F*. It follows

$$F^{\prime }\left(\lambda ,\omega ,y\right)=\left[\begin{array}{ccc} 0 & \Psi^{⊺} & I\\ W\Psi & Z & 0\\ Y & 0 & \Lambda \end{array}\right]$$

where z=Ψλ.

At the *k*th iteration of the algorithm, the Newton step is given by the solution to the nonsingular linear system

(A10) $$F\left(\lambda^{k},w^{k},y^{k}\right)+F^{\prime }\left(\lambda^{k},w^{k},y^{k}\right)*{\left[\Lambda^{k},W^{k},Y^{k}\right]}^{⊺}={\left[e_{m},e_{n},\mu^{k}e_{m}\right]}^{⊺}$$

where y is constrained to satisfy the first KKT condition $y^{k}=e_{m}-\Psi^{⊺}w^{k}$.

The above set of equations can be reduced by standard techniques. It follows:

(A11) $$\begin{array}{cc} \Delta w & =H^{-1}r_{2} \end{array}$$

(A12) $$\begin{array}{cc} \Delta y & =-\Psi \Delta \omega \end{array}$$

(A13) $$\begin{array}{cc} \Delta \lambda & =r_{1}-\lambda -D_{1}\Delta y \end{array}$$

where $H=D_{2}-\Psi D_{1}\Psi^{⊺}$, $D_{2}=ZW^{-1}$, $D_{1}=\Lambda Y^{-1}$, $r_{1}=\mu Y^{-1}e$, $r_{2}=W^{-1}e-\Psi r_{1}$ where the superscript *k* is suppressed for simplicity.

### Appendix A.3. The Algorithm

To describe the algorithm, we need to define the variables: $q=\frac{1}{m}\sum_{i=1}^{m}\lambda_{i}y_{i}$$\rho ={∥e-WZe∥}_{\infty }$ and the scaled duality gap $\gamma =\frac{\left|\Phi (\omega )+\Phi (\Psi \lambda )\right|}{1+|\Phi (\Psi \lambda )|}$.

#### Appendix A.3.1. Initialization

Initially choose $\lambda^{0}=e_{m}/m$, $w^{0}=e_{n}/\Psi \lambda^{0}$, and $y^{0}=e_{m}-\Psi^{⊺}w^{0}$. (Division of two vectors is performed component-wise.) Set $\varepsilon =10^{-8}$.

#### Appendix A.3.2. Iteration

At iteration k+1, set

$$\mu^{k+1}=\sigma^{k}q^{k}$$

where the reduction factor σ is defined by

$$\sigma =\left\{\begin{array}{cc} 1 & ,\mathrm{if}\;\mu \leq \varepsilon \;\mathrm{and}\;\rho >\varepsilon ,\\ \min\left(0.3,{\left(1-\delta_{1}\right)}^{2}\right),{\left(1-\delta_{2}\right)}^{2},\frac{\left|\rho -\mu \right|}{\rho +100\tau } & ,\mathrm{otherwise}. \end{array}\right.$$

The next iterates are given by $\lambda^{k+1}=\lambda^{k}+\delta_{1}\left[\Delta \lambda^{k}\right]$, $\omega^{k+1}=\omega^{k}+\delta_{2}\left[\Delta \omega^{k}\right]$ and $y^{k+1}=y^{k}+\delta_{2}\left[\Delta y^{k}\right]$, where the “damping” factors $\delta_{1}$ and $\delta_{2}$ are defined by

$$\begin{array}{cc} \delta_{1,0} & =-{\left[\min(\min\left(\Lambda^{-1}\Delta \lambda \right),-\frac{1}{2})\right]}^{-1}\\ \delta_{2,0} & =-{\left[\min(\min\left(Y^{-1}\Delta y\right),\min\left(W^{-1}\Delta w\right),-\frac{1}{2})\right]}^{-1}\\ \delta_{1} & =\min(1,0.99995\delta_{1,0})\\ \delta_{2} & =\min(1,0.99995\delta_{2,0}) \end{array}$$

#### Appendix A.3.3. Exit Conditions

Iterate Equations (A11)–(A13) until μ≤ε and ρ≤ε and γ≤ε.

If these conditions are not satisfied after a set number of iterations, then write “PDIP did not converge in the given number of iterations”.

**Table A1:** Simulation versus optimization. Row 1: True simulated means for each parameter. Row 2: NPAG estimates of corresponding means.

	Ka	Vc	Ke	Kcp	Kpc
$\mu_{SIM}$	0.7948080	1.1982732	1.3162403	1.9700861	0.2028168
$\mu_{NPAG}$	0.8003521	1.2054767	1.3108326	1.9780514	0.2059419

**Table A2:** Parameter Covariance Matrices.

Simulation	Ka	V	Ke	Kcp	Kpc
Ka	0.0215919795				
V	0.0011916465	0.0468421011			
Ke	−0.0015398993	−0.0005839048	0.1563336061		
Kcp	0.0009717487	−0.0032613149	−0.0019679155	0.1391046100	
Kpc	−0.0000572936	0.0004080801	0.0007402169	0.0004695783	0.0013055173
**NPAG**	**Ka**	**V**	**Ke**	**Kcp**	**Kpc**
Ka	0.0241232043				
V	0.0038827461	0.0537290654			
Ke	−0.0009243608	−0.0075706783	0.1686441632		
Kcp	−0.0017343788	−0.0083046624	−0.0022665464	0.1617627081	
Kpc	0.0006769795	0.0005601072	0.0027358931	0.0007782649	0.0021142178
